# ZNF468/AURKA/PI3K/AKT Positive Feedback Loop Promotes Proliferation and Metastasis of Oesophageal Squamous Cell Carcinoma

**DOI:** 10.1111/jcmm.70724

**Published:** 2025-07-24

**Authors:** Ge Bai, Lei Wang, Li Zhang, Mayinur Eli

**Affiliations:** ^1^ Cancer Center, The First Affiliated Hospital of Xinjiang Medical University, State Key Laboratory of Pathogenesis, Prevention and Treatment of High Incidence Diseases in Central Asia Xinjiang Medical University Ürümqi China

**Keywords:** AURKA, EMT, ESCC, feedback loop, phosphorylation, PI3K/AKT, ZNF468

## Abstract

While prior research linked ZNF468 to radioresistance in oesophageal squamous cell carcinoma (ESCC), its broader role in ESCC progression remained unclear. This study elucidates these functions and underlying mechanisms. Immunohistochemistry on clinical ESCC tissues demonstrated ZNF468 upregulation, which correlated with unfavourable patient outcomes and increased Aurora A expression. In vitro experiments, including assessments of proliferation, apoptosis, migration and invasion, revealed that ZNF468 overexpression enhanced these oncogenic phenotypes in ESCC cells, while its knockdown produced inhibitory effects. These findings were corroborated in vivo using subcutaneous tumour and lung metastasis models. Mechanistically, ZNF468 was found to upregulate AURKA expression, subsequently activating the PI3K/AKT signalling pathway, thereby promoting cell proliferation and epithelial‐mesenchymal transition (EMT). Importantly, pharmacological inhibition of AURKA or the PI3K/AKT pathway significantly attenuated the pro‐tumorigenic effects driven by ZNF468. Furthermore, AKT was shown to augment ZNF468 protein stability and transcriptional activity, establishing a ZNF468/AURKA/PI3K/AKT positive feedback loop. In conclusion, this study identifies a critical positive feedback mechanism involving ZNF468/AURKA/PI3K/AKT that significantly promotes ESCC progression, underscoring ZNF468 as a potential therapeutic target.

## 1 Introduction

Oesophageal squamous cell carcinoma (ESCC) constitutes a considerable health challenge worldwide, attributable to its high incidence and fatality rates, with limited therapeutic options for advanced stages [1]. The molecular underpinnings of ESCC progression are complex, involving a delicate interplay of genetic and epigenetic alterations driving tumorigenesis, metastasis and resistance to therapies like radiotherapy [2, 3]. Understanding the molecular intricacies of ESCC is vital for devising new therapeutic approaches and identifying prognostic markers. Our previous research identified the role of ZNF468 [4], a zinc finger protein with emerging significance in various cancer types, in conferring radiotherapy resistance in ESCC. We reported that ZNF468 counteracts radiation‐induced G2/M checkpoint arrest and cell death in ESCC by enhancing AURKA gene expression, thereby unveiling a pivotal mechanism by which cancer cells evade the cytotoxic effects of radiotherapy [5]. However, the broader implications of the ZNF468–AURKA axis in ESCC tumorigenesis and its interaction with critical signalling pathways remain unclear.

The PI3K/AKT signalling pathway is critical in regulating cellular functions that enhance cell survival, growth and metastasis, and its dysregulation is frequently implicated in cancer [6]. AKT, a downstream effector of PI3K, phosphorylates numerous substrates regulating cell cycle inhibitors like GSK‐3β and p21, leading to cell cycle progression and enhanced survival [7]. AKT activation promotes cell motility and invasion by modulating matrix metalloproteinases (MMPs), which degrade extracellular matrix components, facilitating cancer cell movement. Additionally, the pathway influences epithelial‐mesenchymal transition (EMT), endowing cells with a more migratory and invasive phenotype marked by a decrease in cellular adhesion factors and an acquisition of mesenchymal characteristics [8]. Studies have demonstrated that AKT regulates the stability and activity of numerous transcription factors [9, 10, 11, 12, 13]. As reported, AKT phosphorylates ZNF322A at Thr‐262 in lung cancer and promotes cancer progression [14]. AKT can also phosphorylate Sox2 at threonine 118 (Thr, T), preventing the methylation of lysine 119 and subsequent Sox2 degradation [15]. Additionally, in cases of HER2 overexpression, AKT phosphorylates HSF‐1 at serine 326 (Ser326), enhancing Slug promoter activity and thereby inducing breast cancer cell metastasis [16]. The phosphorylation by AKT at Thr258 of Prohibitin 1 promotes the proliferation of bladder cancer [17]. AKT‐mediated phosphorylation of Sp1 at serines 42, threonines 679 and serines 698 induces CCR7 expression following COX‐2 stimulation [13]. These findings suggest that the phosphorylation of transcription factors by AKT, which modulates their DNA binding affinity, transcription complex assembly and protein stability, is associated with cancer progression.

Aurora A, a key mitotic regulator, interfaces with the PI3K/AKT pathway, amplifying its oncogenic signals [18, 19]. Aurora A overexpression enhances AKT activity, creating a feedforward loop that sustains cancer cell malignancy and promotes centrosome amplification and chromosomal instability, contributing to tumour progression by increasing the genetic diversity of the tumour cell population, potentially enhancing adaptability to therapeutic interventions. Aurora A is also involved in EMT, where it phosphorylates and inactivates proteins such as Rb, leading to decreased E‐cadherin expression and increased N‐cadherin expression [20]. Crosstalk between Aurora A and PI3K/AKT signalling drives tumour cell proliferation, invasion and metastasis through multifaceted mechanisms [19]. Given the established role of PI3K/AKT in ESCC and its potential crosstalk with the ZNF468–AURKA axis, this study elucidates how the ZNF468–AURKA axis collaborates with PI3K/AKT signalling to drive tumorigenesis and EMT in ESCC, revealing a novel regulatory mechanism and potential therapeutic target.

## 2 Materials and Methods

### 2.1 Patients and Specimens

Tumour tissues and matched non‐tumour tissues from 58 patients with ESCC were retrospectively identified for this study. These samples were originally collected during routine clinical surgical procedures at The First Affiliated Hospital of Xinjiang Medical University between March 2022 and April 2024. Inclusion criteria included histologically confirmed ESCC, TNM stages I–III and no prior anticancer therapy. Following surgical resection, tissues were flash‐frozen in liquid nitrogen and stored at −80°C. Written informed consent, which included permission for the future use of tissue samples in research, was obtained from all participants at the time of their initial surgery and sample collection. This study was approved by the ethics committee of the First Affiliated Hospital of Xinjiang Medical University (approval no. 20220308‐091).

### 2.2 Bioinformatics Analysis

Analysis of the expression differences and clinical correlations of ZNF468 and AURKA across multiple public datasets was performed using the BEST bioinformatics analysis web tool [21].

### 2.3 Cell Culture

The KYSE510 and KYSE140 cell lines, obtained from the Chinese Academy of Sciences Type Culture Collection, were cultured in Dulbecco's Modified Eagle Medium (DMEM; Corning) supplemented with 10% fetal bovine serum (FBS) and maintained at 37°C in a humidified 5% CO_2_ atmosphere. KYSE510 and KYSE140 cell lines were chosen for this study as they are established and widely used in vitro models for investigating the biology of ESCC [5, 22, 23].

### 2.4 Plasmids and Cell Transfection

For the overexpression and knockdown methods of ZNF468, please refer to our previous study [5]. Overexpression of AURKA was achieved by inserting the complete AURKA coding sequence into the pLV‐EGFP:T2A:Puro‐EF1A vector. Conversely, AURKA was knocked down using shRNA oligos, which were incorporated into the pLKO.1 vector. The target sequence for shAURKA was 5′‐GCATTTCAGGACCTGTTAAGG‐3′ and a scrambled shRNA (SHC002 Sigma‐Aldrich; 5′‐CCGG‐CAACAAGATGAAGAGCACCAA‐CTCGAG‐TTGGTGCTCTTCATCTTGTTG‐TTTTT‐3′) was used as a non‐specific control. Cell transfection was conducted with Lipofectamine 2000.

### 2.5 Immunohistochemistry (IHC)

Tissue sections from 58 ESCC patients were processed for IHC to evaluate protein expression levels. Following antigen retrieval and blocking procedures, samples were incubated with primary antibodies as listed (Table 1), followed by application of a peroxidase‐conjugated secondary antibody. Chromogenic substrate development with DAB visualised specific protein binding. Slides were counterstained, dehydrated and examined under a microscope. Protein expression was quantified using the average optical density (MOD) value.

**TABLE 1:** The antibodies used in this study.

Reagent	Company	Catalogue number	Dilution
Anti‐ZNF468 (WB)	Abcam	ab88862	1:2000
Anti‐ZNF468 (IHC)	Invitrogen	#PA5‐68447	1:150
Anti‐Aurora A (WB)	CST	#14475S	1:1000
Anti‐Aurora A (IHC)	CST	# 91590T	1:250
Anti‐Bax (WB)	CST	#2772S	1:1000
Anti‐Bcl‐2 (WB)	CST	#3498S	1:1000
Anti‐cleaved caspase 3 (WB)	CST	#9664S	1:1000
Anti‐GAPDH (WB)	CST	#2118S	1:1000
Anti‐PI3K p85 (WB)	CST	#4292	1:1000
Anti‐phos‐PI3K p85 (WB)	Thermo fisher	PA5‐104853	1:2000
Anti‐AKT (WB)	Genetex	GTX121937	1:1000
Anti‐phos‐AKT (WB)	Genetex	GTX128414	1:500
Anti‐E‐Cadherin (WB)	CST	3195S	1:1000
Anti‐N‐Cadherin (WB)	CST	13116S	1:1000
Anti‐Snail (WB)	CST	3879S	1:1000
Anti‐ZO‐1 (WB)	CST	13663S	1:1000
Anti‐Bcl2 (IF)	Thermo fisher	MA5‐41210	1:200
Anti‐Ki67 (IF)	Thermo fisher	MA5‐14520	1:500
HA tag (WB)	GeneTex	GTX29110	1:2000
GST tag (WB)	GeneTex	GTX110736	1:3000
GFP tag (WB)	GeneTex	GTX26556	1:5000
Phospho‐(Ser/Thr) Akt substrate	CST	#9611	1:1000

*Note:* All antibodies not specifically labelled were used for western blot.

### 2.6 Western Blot (WB)

Proteins were extracted from cells or tissues utilising a commercial protein extraction kit (ab270054, Abcam). Subsequently, proteins were separated by SDS‐PAGE, transferred onto PVDF membranes, blocked with a 5% skim milk solution and incubated with primary antibodies at 4°C overnight, prior to incubation with the secondary antibodies.

### 2.7 Colony Formation Assay

Cells of the KYSE510 or KYSE140 lines were plated at a density of 500 cells per well in 6‐well plates and cultured for 14 days. Subsequently, the plates were treated with methanol for a 10‐min fixation and then stained with crystal violet for 30 min. Once the staining process was complete, the colonies were enumerated and subjected to analysis.

### 2.8 EdU Staining

KYSE510 and KYSE140 cells were plated into 96‐well plates. Subsequently, the culture medium was supplemented with EdU reagent, and the incubation was extended. After incubation, the cells were fixed using a 4% paraformaldehyde solution for 10 min. Thereafter, the cells underwent treatment with an EdU conjugation buffer. Following treatment, the cells were washed to remove unreacted reagent and imaged using a fluorescence microscope.

### 2.9 Wound Healing

KYSE510 and KYSE140 cells were plated into 6‐well plates and cultured to confluence. A sterile pipette tip was employed to uniformly wound the cell monolayer by scraping. Subsequently, detached cells and debris were removed by washing gently with phosphate‐buffered saline (PBS). The cells were then incubated with fresh culture medium containing a reduced concentration of fetal bovine serum (1% FBS) to minimise confounding effects from cell proliferation while permitting cell migration. The process of wound closure was then observed over time using a microscope, with images captured at 0 h and 24 h post‐wounding. The distance of wound closure was measured.

### 2.10 Transwell Assay

KYSE510 and KYSE140 cells were suspended in serum‐free medium and subsequently plated into the upper chamber of a Transwell insert, either uncoated for migration or coated with Matrigel for invasion. To establish a chemoattractant gradient, complete culture medium containing 10% fetal bovine serum (FBS) was added to the lower chamber. Following incubation, cells that had migrated or invaded through the membrane were then fixed and stained. Subsequently, the stained cells were quantified using a microscope to determine migration and invasion rates.

### 2.11 Animal Model

Cell line‐derived xenograft (CDX) model was developed by subcutaneously injecting 6‐week‐old male BALB/c nude mice using KYSE510 cells. The mice were subsequently allocated into three separate groups, each comprising six mice and received a subcutaneous injection of 2 × 10^6^ KYSE510 cells per mouse. Starting from day 7, the volume of subcutaneous tumours was monitored every 3 days until day 19. The tumour tissues were then excised, weighed and subjected to subsequent experiments. For in vivo metastasis model, 8‐week‐old female BALB/c nude mice were maintained in accordance with institutional protocols. A suspension of ESCC cells, containing 2 × 10^6^ cells for each mouse, was injected intravenously through the tail vein. Bioluminescent imaging (Xenogen IVIS lumima II, PerkinElmer, MA) was conducted 9 weeks post‐cell injection to monitor metastasis. Then, the animals were euthanised, and their lungs were extracted for further examination. The BALB/c nude mice in this study were housed in individually ventilated cages under controlled temperature, humidity and a 12‐h light/dark cycle, with ad libitum access to standard chow and water. Euthanasia was performed by CO_2_ inhalation using a gradual volume displacement rate of 30% per minute. After the mice became unconscious and ceased movement, the CO_2_ flow rate was increased to ensure complete saturation of the chamber atmosphere. Death was confirmed by verifying the complete cessation of respiration and movement, along with pupillary dilation. Following this confirmation, the CO_2_ supply was discontinued, and the animals were observed for three additional minutes to ensure euthanasia was irreversible.

### 2.12 Immunofluorescent Staining

CDX tumour tissue sections from nude mice were processed for dual immunofluorescent staining of KI67 and BCL2. After fixation and permeabilisation, tissue sections were blocked and incubated with a mouse monoclonal anti‐KI67 antibody. Subsequently, the sections were treated with a rabbit polyclonal anti‐BCL2 antibody and visualised with a fluorophore‐conjugated secondary antibody of a different wavelength. Nuclei were counterstained with DAPI.

### 2.13 Haematoxylin and Eosin (HE) Staining

CDX tumour tissues were processed for HE staining to evaluate histopathological features. Sections were fixed, sectioned and stained with Haematoxylin to highlight cell nuclei and eosin to identify cytoplasmic structures. The stained slides were examined microscopically to assess tumour morphology and tissue architecture.

### 2.14 Cycloheximide (CHX) Chase Assay

KYSE510 cells were transfected using a plasmid encoding an HA‐tagged ZNF468 for 24 h, followed by treatment with CHX at 20 μg/mL for varying durations. Protein lysates were harvested at specified time points, and protein levels were assessed by WB.

### 2.15 Dual Luciferase Promoter Assay

Gene promoter activity was assessed using the Dual‐Luciferase Reporter Assay System (Promega, Madison, WI, USA), with data expressed as the mean ratio of firefly luciferase to Renilla luciferase activity, and experiments were performed in triplicate.

### 2.16 AKT In Vitro Kinase assay

GST‐tagged ZNF468 protein (1 μg) was incubated with escalating concentrations of active full‐length recombinant AKT protein. The reaction occurred in a kinase buffer containing 0.5 mM unlabelled ATP and 5 nM γ‐ATP‐^32^P at 30°C for 30 min. To terminate the reactions, an equal volume of 2X SDS loading buffer was added, followed by heating at 95°C for 5 min. The samples were then resolved by 8% SDS‐PAGE, transferred to nitrocellulose membranes and analysed using autoradiography or WB.

### 2.17 Statistics

Data were expressed as mean ± SD and analysed using one‐way ANOVA or the Wilcoxon test with GraphPad Prism 9.5. A *p* value of < 0.05 was considered statistically significant.

## 3 Results

### 3.1 High ZNF468 Expression Levels in ESCC Are Linked to Adverse Clinical Outcomes and Strongly Correlate With Aurora A Expression

First, using the BEST bioinformatics analysis web tool [21], our findings revealed that ZNF468 mRNA levels were elevated in cancer groups across three independent cohorts (GSE53622, GSE53624, TCGA‐ESCA) (Figure S1A). In the TCGA‐ESCA cohort, elevated ZNF468 mRNA expression was correlated with higher pathological grades and lymph node infiltration (Figure S1B), indicating the close association with the progression of ESCC. Additionally, as the gene encoding the well‐known oncogenic protein Aurora A, AURKA is significantly upregulated in tumour tissues across multiple datasets (GSE104958, GSE130078, GSE52622, GSE53624, TCGA‐ESCA) (Figure S1C). Our prior investigation identified AURKA as a downstream target of ZNF468 [5]. We performed IHC on tissue samples from 58 ESCC patients, categorising patients into high and low ZNF468 or Aurora A expression groups based on the median mean optical density (MOD) value. Chi‐square tests revealed that patients in the high‐expression groups for ZNF468 or Aurora A had significantly higher T stages, increased lymph node infiltration and more frequent tumour metastasis (Table 2 and Table 3). ZNF468 and Aurora A protein levels were significantly elevated in cancerous tissue versus adjacent benign tissue (Figure S1D,E). Our analysis using Pearson correlation identified a significant positive relationship (*R* = 0.53) between the expression levels of ZNF468 and Aurora A protein (Figure S1F).

**TABLE 2:** Analysis of clinical characteristic differences between high and low ZNF468 expression groups in ESCC patients.

	Low (*N* = 29)	High (*N* = 29)	*p*
Age			
≥ 65	16 (55.2%)	18 (62.1%)	0.79
< 65	13 (44.8%)	11 (37.9%)	
Gender			
Male	13 (44.8%)	15 (51.7%)	0.793
Female	16 (55.2%)	14 (48.3%)	
Stage			
I	4 (13.8%)	2 (6.9%)	0.0302
II	18 (62.1%)	9 (31.0%)	
III	5 (17.2%)	10 (34.5%)	
IV	2 (6.9%)	8 (27.6%)	
T			
T1	0 (0%)	1 (3.4%)	0.0274
T2	4 (13.8%)	1 (3.4%)	
T3	25 (86.2%)	21 (72.4%)	
T4	0 (0%)	6 (20.7%)	
N			
N0	22 (75.9%)	12 (41.4%)	0.0263
N1	3 (10.3%)	9 (31.0%)	
N2	4 (13.8%)	8 (27.6%)	
M			
M0	27 (93.1%)	21 (72.4%)	0.0822
M1	2 (6.9%)	8 (27.6%)	

**TABLE 3:** Analysis of clinical characteristic differences between high and low AURKA expression groups in ESCC patients.

	Low (*N* = 29)	High (*N* = 29)	*p*
Age			
≥ 65	15 (51.7%)	19 (65.5%)	0.424
< 65	14 (48.3%)	10 (34.5%)	
Gender			
Male	15 (51.7%)	13 (44.8%)	0.793
Female	14 (48.3%)	16 (55.2%)	
Stage			
I	4 (13.8%)	2 (6.9%)	0.0448
II	16 (55.2%)	11 (37.9%)	
III	8 (27.6%)	7 (24.1%)	
IV	1 (3.4%)	9 (31.0%)	
T			
T1	0 (0%)	1 (3.4%)	0.135
T2	4 (13.8%)	1 (3.4%)	
T3	24 (82.8%)	22 (75.9%)	
T4	1 (3.4%)	5 (17.2%)	
N			
N0	21 (72.4%)	13 (44.8%)	0.0737
N1	5 (17.2%)	7 (24.1%)	
N2	3 (10.3%)	9 (31.0%)	
M			
M0	28 (96.6%)	20 (69.0%)	0.015
M1	1 (3.4%)	9 (31.0%)	

### 3.2 ZNF468 Promotes ESCC Cell Proliferation and Anti‐Apoptosis Partly Mediated by Upregulating AURKA In Vitro

**FIGURE 1:**
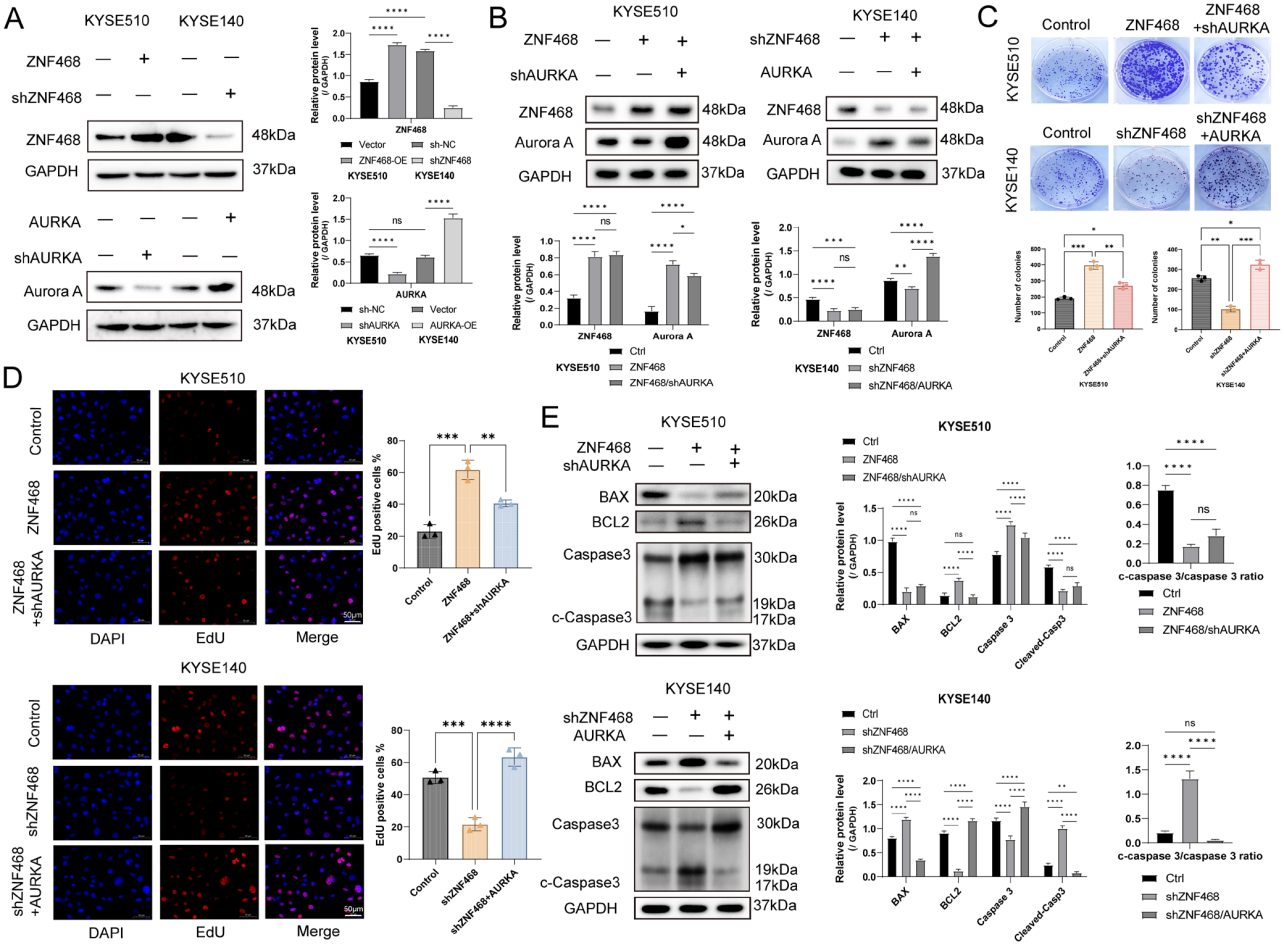
ZNF468 promotes ESCC cell proliferation and anti‐apoptosis partly mediated by upregulating AURKA in vitro. (A) Western blot analysis was performed to assess the transfection efficiency of ZNF468 or AURKA knockdown and overexpression in KYSE510 and KYSE140 cell lines. (B) Western blot analysis of the protein expression levels of ZNF468 and Aurora A after AURKA knockdown in ZNF468‐overexpressing KYSE510 cells and after AURKA overexpression in ZNF468‐knockdown KYSE140 cells. (C) Representative images and statistical analysis of the clone formation assay 2 weeks after AURKA knockdown in ZNF468‐overexpressing KYSE510 cells, and after AURKA overexpression in ZNF468‐knockdown KYSE140 cells (*n* = 3 biological replicates). (D) Proliferation capacities assessed by EdU staining assay and statistical analysis (*n* = 3 biological replicates). Scale bar: 50 μm. (E) Western blot analysis of the expression levels of apoptosis‐related markers Bax, Bcl2 and cleaved caspase 3. Data were presented as mean ± SD. Ordinary One‐way ANOVA test. *p < 0.05; **p < 0.01; ***p < 0.001; ****p < 0.0001.

In our previous research, we successfully generated KYSE140 cells with ZNF468 knockdown and KYSE510 cells overexpressing ZNF468 [5]. To further elucidate the function of the ZNF468–AURKA axis, we knocked down AURKA in KYSE510 cells with ZNF468 overexpression and overexpressed AURKA in KYSE140 cells with ZNF468 knockdown. Western blot analysis was performed to assess the transfection efficiency of ZNF468 or AURKA knockdown and overexpression in KYSE510 and KYSE140 cell lines (Figure 1A). WB analysis demonstrated that elevated ZNF468 levels enhanced Aurora A protein expression, while ZNF468 knockdown inhibited Aurora A protein expression. However, overexpression or knockdown of Aurora A did not affect ZNF468 expression levels (Figure 1B). Colony formation and EdU incorporation assays revealed that ZNF468 overexpression markedly boosted ESCC cell proliferation, whereas this effect was notably inhibited upon Aurora A knockdown. Conversely, ZNF468 knockdown significantly suppressed ESCC cell proliferation, but overexpression of Aurora A restored the proliferative capacity of the cells (Figure 1C,D). Next, we assessed apoptosis‐associated proteins using WB. The findings indicated that ZNF468 overexpression markedly increased BCL2 levels, while decreasing BAX and cleaved caspase‐9 expression. However, when AURKA was knocked down, the anti‐apoptotic effects induced by ZNF468 overexpression were markedly attenuated. Conversely, knockdown of ZNF468 markedly decreased BCL2 levels and concurrently elevated those of BAX and cleaved caspase‐3. However, under conditions of AURKA overexpression, the anti‐apoptotic capacity of ESCC cells was substantially restored (Figure 1E). These findings indicate that ZNF468 promotes ESCC cell proliferation and anti‐apoptosis, partly mediated by upregulating AURKA in vitro.

### 3.3 ZNF468–AURKA Axis Promotes ESCC Cell Migration and Invasion by Enhancing EMT

**FIGURE 2:**
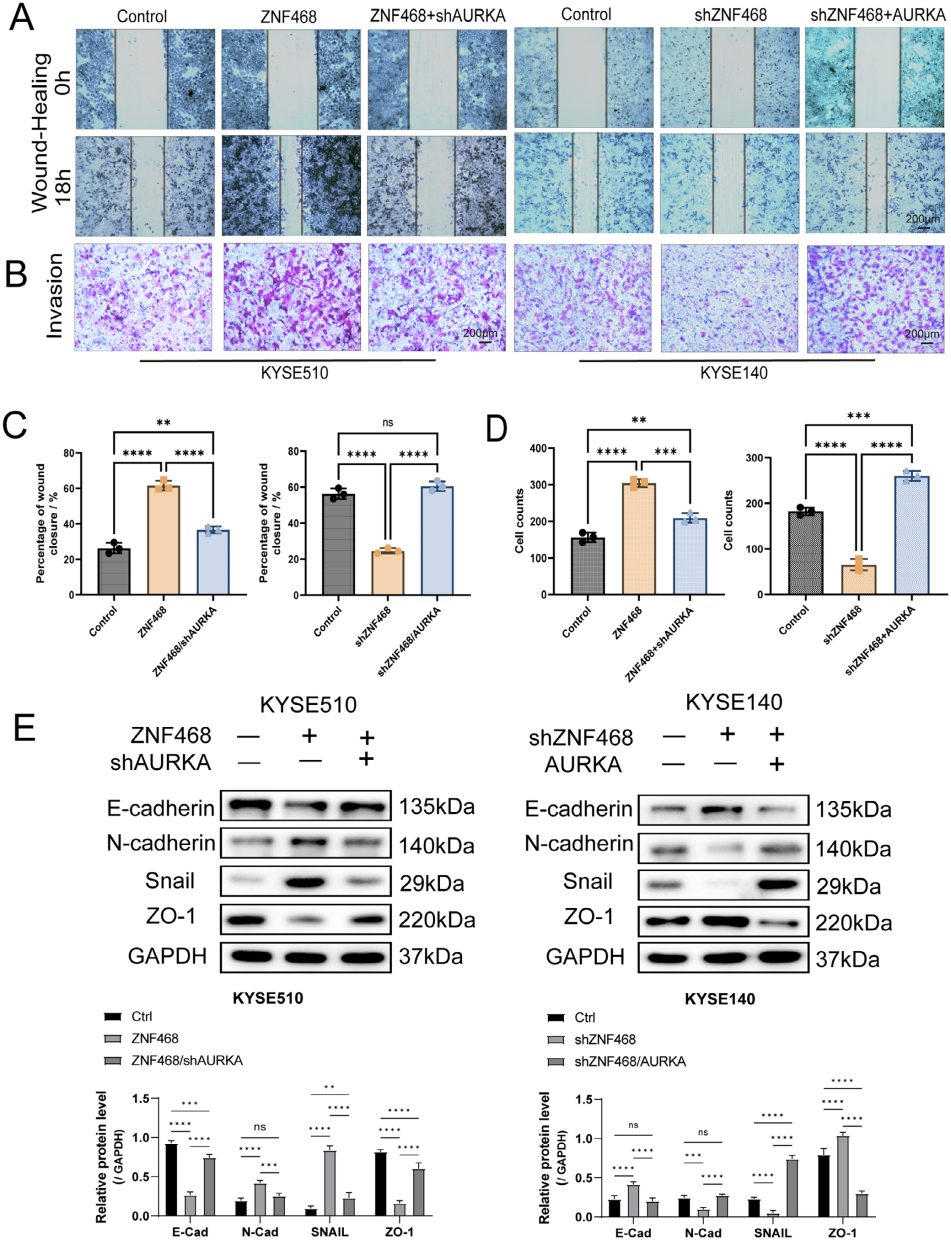
ZNF468‐AURKA axis promotes ESCC cell migration and invasion by enhancing epithelial‐mesenchymal transition in vitro. (A) Representative images of the wound‐healing assay at 0 and 18 h after AURKA knockdown in ZNF468‐overexpressing KYSE510 cells and after AURKA overexpression in ZNF468‐knockdown KYSE140 cells (*n* = 3 biological replicates). Scale bar: 200 μm. (B) Invasion abilities assessed by Transwell assay after AURKA knockdown in ZNF468‐overexpressing KYSE510 cells and after AURKA overexpression in ZNF468‐knockdown KYSE140 cells (*n* = 3 biological replicates). Scale bar: 200 μm. (C, D) Statistical analysis results of the percentage of wound closure in the wound‐healing Assay and the number of invading cells in the Transwell invasion assay. (E) Western blot analysis of the expression levels of epithelial‐mesenchymal transition‐related markers N‐Cadherin, Snail, E‐Cadherin and ZO‐1. Data were presented as Mean ± SD. One‐way ANOVA test. ns: not significant, **p < 0.01; ***p < 0.001; ****p < 0.0001.

Given that ZNF468 is highly correlated with tumour metastasis and lymph node infiltration in clinical samples, we subsequently explored the function of the ZNF468–AURKA axis in ESCC cell invasion and migration. As expected, wound‐healing assays showed that ZNF468 overexpression enhanced ESCC cell migration, while ZNF468 knockdown exerted the opposite. Moreover, the enhancing effect of ZNF468 on migration was significantly inhibited when AURKA was knocked down (Figure 2A,C). Consistently, Transwell invasion assays demonstrated that ZNF468 mediated ESCC cell invasion via AURKA (Figure 2B,D). EMT is recognised as a crucial driver of cancer cell migration and invasion [24]. While some studies have shown that Aurora A can induce EMT in certain cancers, its role in EMT in ESCC remains unexplored [20, 25, 26]. Therefore, we assessed EMT marker expression via WB. The findings indicated that ZNF468 overexpression markedly increased N‐Cadherin and Snail protein levels, while downregulating E‐Cadherin and ZO‐1. Conversely, ZNF468 knockdown led to opposite effects. Importantly, AURKA knockdown significantly attenuated the ZNF468 overexpression‐induced promotion of EMT, while AURKA overexpression markedly reversed the EMT reduction caused by ZNF468 knockdown (Figure 2E). These findings suggest that the ZNF468–AURKA axis is a key factor in promoting ESCC cell invasion and migration via the regulation of EMT.

### 3.4 The ZNF468–AURKA Axis Activates the PI3K/AKT Signalling Pathway, Thereby Mediating Oncogenic Phenotypes in ESCC

**FIGURE 3:**
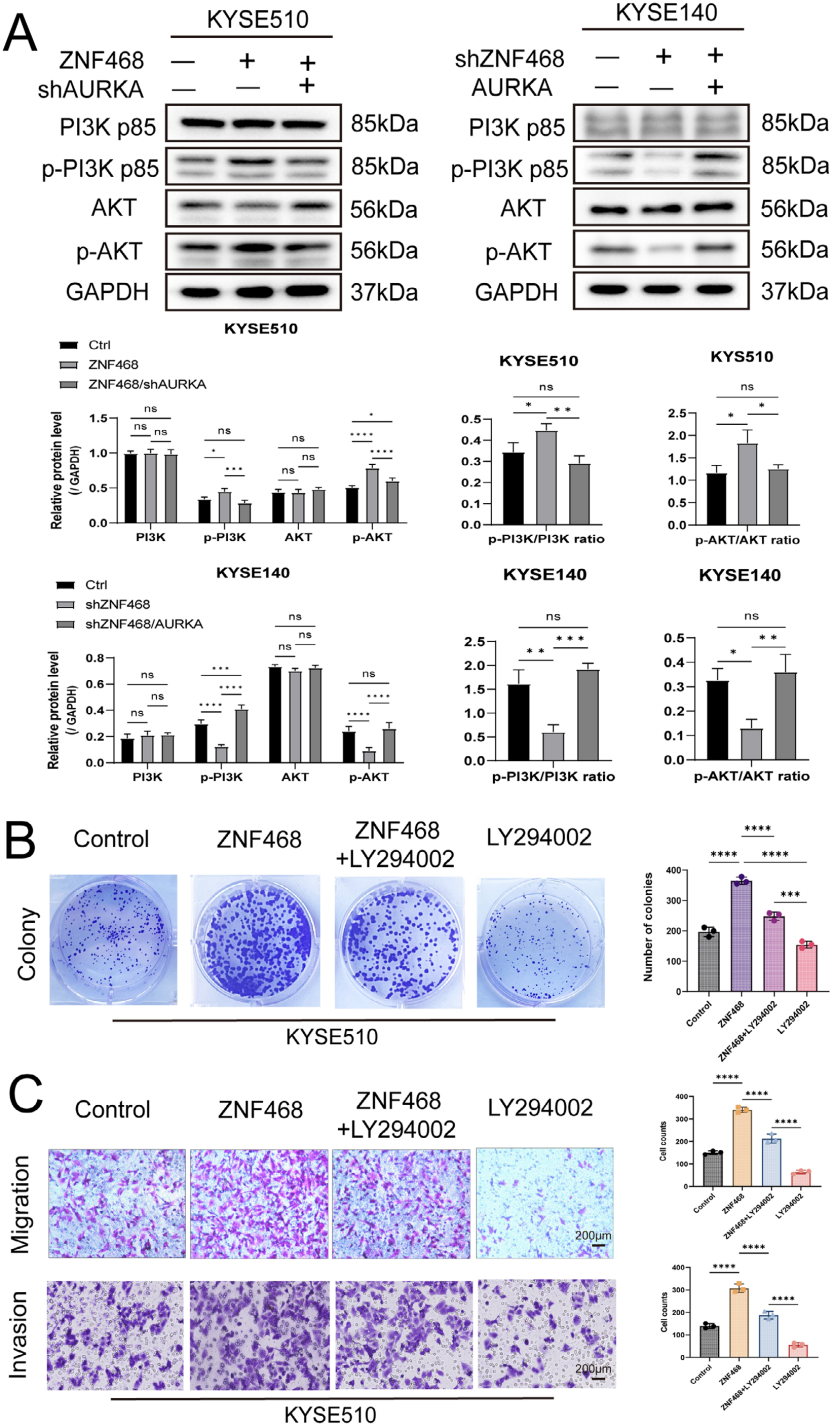
ZNF468‐AURKA mediates ESCC oncogenic phenotypes through activation of the PI3K/AKT signalling pathway. (A) Western blot analysis of the protein expression levels of PI3K, p‐PI3K, AKT and p‐AKT after AURKA knockdown in ZNF468‐overexpressing KYSE510 cells and after AURKA overexpression in ZNF468‐knockdown KYSE140 cells. (B) Representative images and statistical analysis of the clone formation assays with or without PI3K inhibitor LY294002 in ZNF468‐overexpressing KYSE510 cells (*n* = 3 biological replicates). (C) Migration and invasion abilities assessed by Transwell assay with or without PI3K inhibitor LY294002 in ZNF468‐overexpressing KYSE510 cells (*n* = 3 biological replicates). Scale bar: 200 μm. Data were presented as mean ± SD. Ordinary One‐way ANOVA test. ns, not significant, *p < 0.05; **p < 0.01; ***p < 0.001; ****p < 0.0001.

Having established that ZNF468 promotes oncogenic phenotypes through AURKA, we then investigated the molecular mechanisms behind its effects. Aberrant activation of the PI3K/AKT signalling axis critically drives cancer progression by enhancing malignant phenotypes, including uncontrolled proliferation, migratory potential and invasive capacity of tumour cells [6, 8, 27]. Additionally, existing evidence suggests that AURKA promotes PI3K/AKT activities across different cancer types [18, 19, 28]. Especially, AURKA phosphorylates SDCBP, inhibiting its ubiquitination and degradation, thereby maintaining SDCBP protein stability. SDCBP, in turn, engages directly with EGFR, preserving its localisation to the membrane and triggering the EGFR‐PI3K‐AKT signalling cascade in ESCC [18]. Therefore, we first investigated the regulatory relationship between ZNF468 and PI3K/AKT. WB findings revealed ZNF468 overexpression elevated the expression of p‐PI3K and p‐AKT, whereas ZNF468 knockdown had the opposite effect. PI3K and AKT total protein levels remained constant. Furthermore, when ZNF468 was overexpressed and AURKA was knocked down, the expressions of p‐PI3K and p‐AKT were markedly reduced. Conversely, in ZNF468 knockdown cells with AURKA overexpression, the levels of p‐PI3K and p‐AKT were restored. AURKA knockdown or overexpression did not affect the PI3K and AKT total protein levels (Figure 3A). The results indicate that ZNF468 may activate the PI3K/AKT signalling pathway via Aurora A. Subsequently, we treated KYSE510 cells overexpressing ZNF468 with 10 μM of the PI3K inhibitor LY294002 for 48 h. LY294002 is a widely used PI3K inhibitor that specifically inhibits PI3K activity, thereby blocking the PI3K/AKT signalling pathway [29, 30]. The colony formation assay showed that cell proliferation was significantly reduced in the group treated with LY294002 compared to the ZNF468 overexpression group (Figure 3B). Moreover, Transwell assays indicated that ZNF468 overexpression could not significantly promote ESCC cell migration and invasion under LY294002 co‐treatment conditions (Figure 3C). These results confirm that elevated ZNF468–AURKA axis promotes oncogenic phenotypes in ESCC via the PI3K/AKT pathway.

### 3.5 The Elevated ZNF468–AURKA Axis Promotes Subcutaneous Tumour Formation, EMT and Activation of the PI3K/AKT Pathway of ESCC In Vivo

**FIGURE 4:**
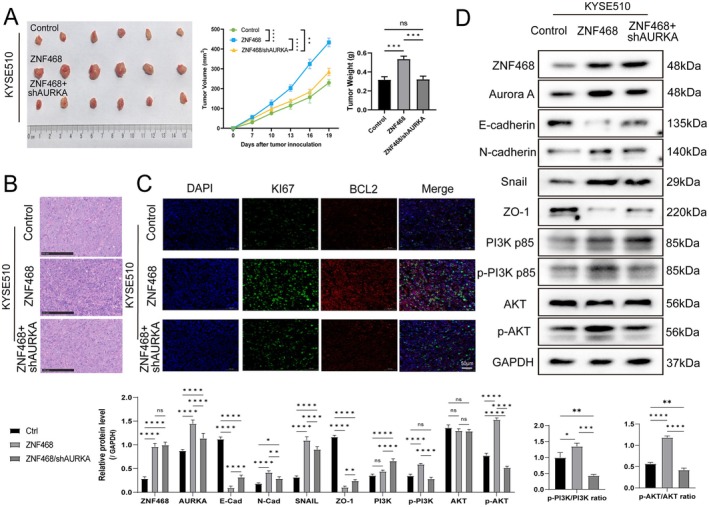
Elevated ZNF468‐AURKA axis promotes subcutaneous tumour formation, epithelial‐mesenchymal transition and activation of PI3K/AKT pathway of ESCC in vitro. (A) Tumours were generated in NOD/SCID mice by subcutaneously injecting each mouse with 1 × 10^7^ KYSE510 cells (*n* = 6 per group). The tumour volumes from Day 0 to Day 19 and tumour weight at Day 19 were subsequently evaluated. (B) Representative haematoxylin and eosin (HE)‐stained pathological images of tumour tissues from the Control, ZNF468 and ZNF468/shAURKA groups. Scale bar: 250 μm. (C) Representative images of KI67 (Green) and BCL2 (Red) immunofluorescence double staining for the Control, ZNF468 and ZNF468/shAURKA groups. Scale bar: 50 μm. (D) Western blot analysis of epithelial‐mesenchymal transition (EMT) and PI3K/AKT pathway‐related markers in tumour tissues from nude mice. Data were presented as Mean ± SD. One‐way ANOVA test. ns: not significant, *p < 0.05; **p < 0.01; ***p < 0.001; ****p < 0.0001.

We conducted a subcutaneous tumorigenesis experiment using KYSE510 cells in BALB/c nude mice. The ZNF468 overexpression group exhibited the highest tumour volume and weight, followed by the ZNF468 overexpression combined with AURKA knockdown group, and the control group had the smallest tumours (Figure 4A). HE staining indicated that cancer tissues from the ZNF468 overexpression group exhibited more condensed nuclei, higher degrees of damage and less distinct cell boundaries. In contrast, the control group displayed the least pathological damage. The ZNF468 overexpression combined with AURKA knockdown group showed significantly reduced malignancy compared to the ZNF468 overexpression group (Figure 4B). Immunofluorescence showed the highest KI67 and BCL2 fluorescence intensity in tumour tissues from the ZNF468 overexpression group. This intensity was significantly reduced in the ZNF468 overexpression combined with AURKA knockdown group, indicating that AURKA knockdown weakened the effects that promote cell proliferation and inhibit apoptosis of ZNF468 (Figure 4C). Subsequently, we employed WB analysis to assess the expression of EMT markers and PI3K/AKT pathway proteins. ZNF468 overexpression significantly upregulated N‐Cadherin and Snail protein levels in tumour tissues, while downregulating E‐Cadherin and ZO‐1. The PI3K/AKT activity exhibited marked upregulation in tumour tissues of the ZNF468 overexpression group, and this activation was markedly reduced following AURKA knockdown (Figure 4D). These in vivo results further demonstrate that ZNF468 upregulates AURKA, promoting ESCC cell proliferation and EMT through activation of PI3K/AKT.

### 3.6 The ZNF468–AURKA Axis Promotes Lung Metastasis of Oesophageal Cancer Cells in a Nude Mouse Model of Tail Vein Pulmonary Metastasis

**FIGURE 5:**
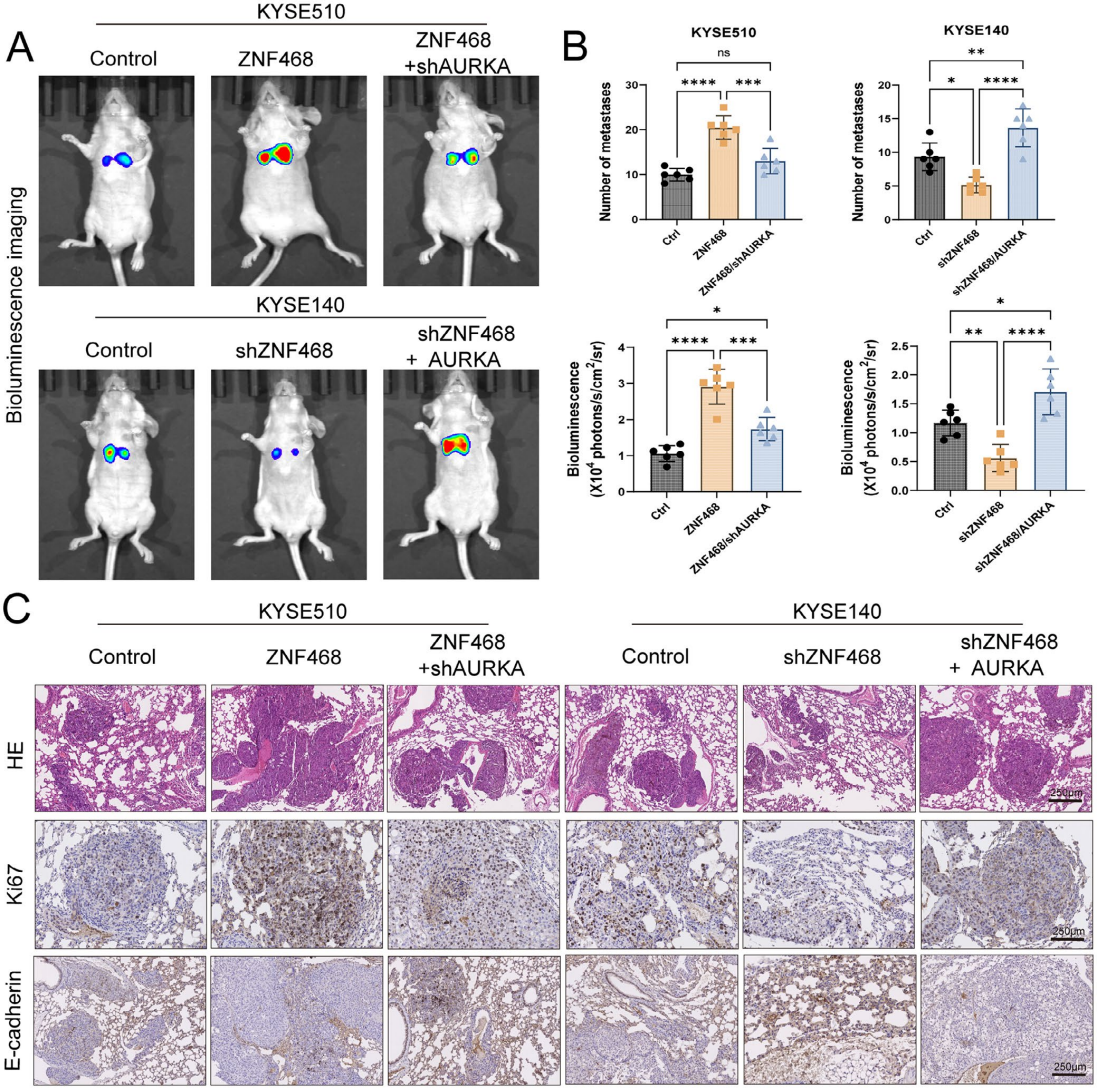
ZNF468‐AURKA axis promotes lung metastasis of oesophageal cancer cells in a nude mouse model of tail vein pulmonary metastasis. (A) Lung metastasis in nude mice, following intravenous injections of ZNF468‐overexpressing KYSE510 or ZNF468‐knockdown KYSE140 cells, was monitored using bioluminescence imaging. (B) Statistical results of the number of metastatic foci and bioluminescence intensity in metastatic tissues. Data are presented as mean ± SD. One‐way ANOVA test was used for analysis. ns: Not significant, *p < 0.05; **p < 0.01; ***p < 0.001; ****p < 0.0001. (C) Representative images of H&E staining and immunohistochemical staining for Ki67 and E‐cadherin in metastatic lung tissues. Scale bar: 250 μm.

To further verify the role of ZNF468 in oesophageal cancer metastasis, a model for experimental metastasis was developed by administering KYSE150‐luc or KYSE140‐luc cells intravenously through the tail vein of nude mice. Monitoring of lung metastasis was achieved using bioluminescence imaging, with confirmation through HE and IHC staining. Bioluminescence imaging results indicated ZNF468 significantly increased the pulmonary metastatic burden of KYSE510 cells in oesophageal cancer, while the pro‐metastatic effect of ZNF468 was markedly diminished under conditions of AURKA knockdown. On the other hand, knockdown of ZNF468 significantly inhibited pulmonary metastasis in KYSE140 cells, and the aforementioned inhibitory effect was significantly reversed under conditions of AURKA overexpression (Figure 5A,B). Pathological staining results from H&E and IHC also confirmed the aforementioned trends. ZNF468 overexpression significantly increased the tumour size, number of metastatic foci and malignancy of pulmonary metastases, with a marked increase in the staining intensity of the proliferation marker Ki67 and a marked reduction in the levels of the EMT marker E‐cadherin (Figure 5C). Conversely, knockdown of ZNF468 had the opposite effect, indicating ZNF468 is a promising therapeutic target for inhibiting pulmonary metastasis in oesophageal cancer.

### 3.7 The Phosphorylation of AKT Enhances Protein Stability of ZNF468 and Transcription Activity of ZNF468 on AURKA

**FIGURE 6:**
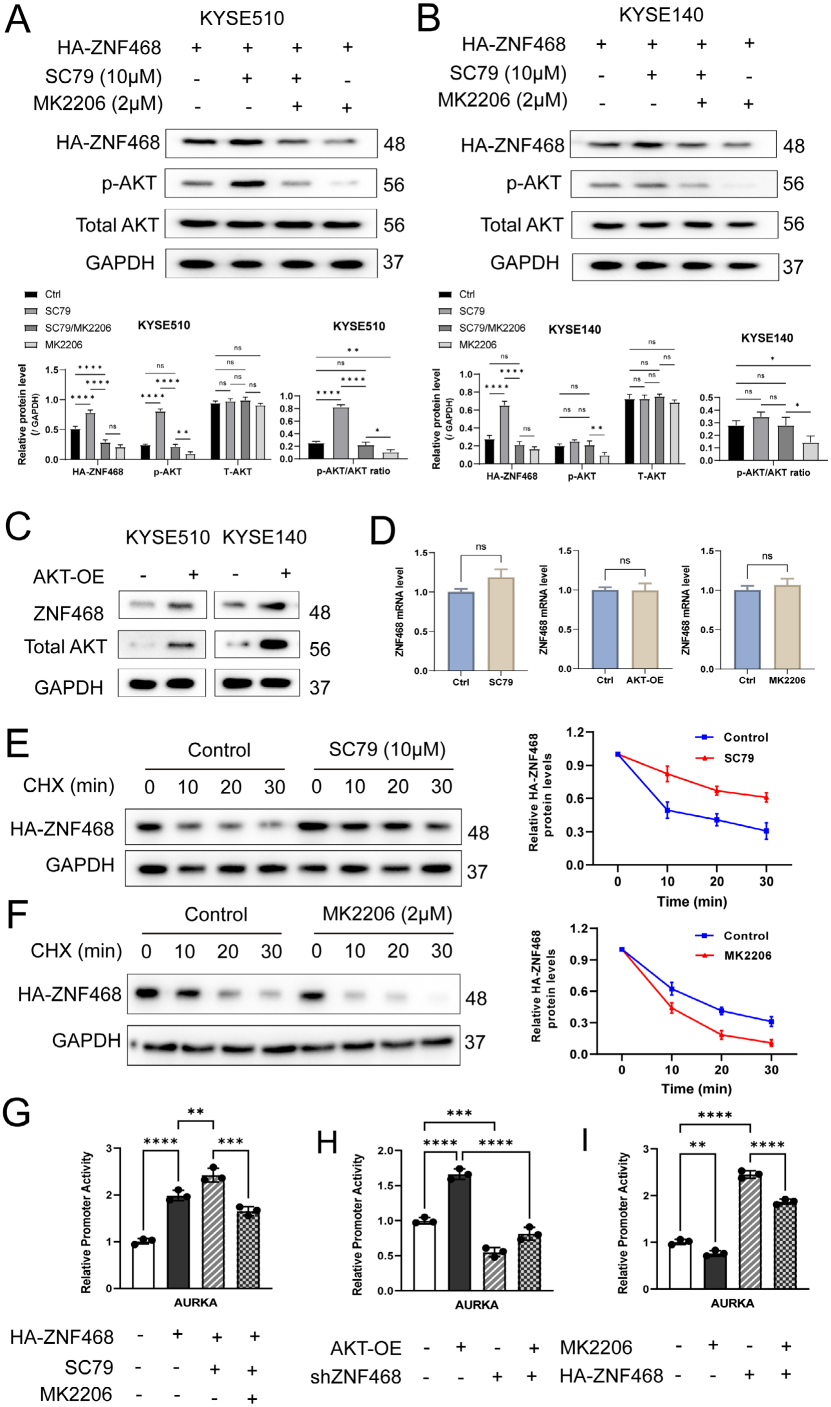
The phosphorylation of AKT enhances protein stability of ZNF468 and transcription activity of ZNF468 on AURKA. (A, B) Western blotting was performed on lysates from KYSE510 and KYSE140 cells that expressed HA‐ZNF468. Following a 12‐h serum deprivation, cells were incubated with AKT activator SC79 (10 μM) alone or in conjunction with AKT inhibitor MK2206 (2 μM) for 3 h. (C) Immunoblotting assessed endogenous ZNF468 levels in AKT‐overexpressing KYSE510 and KYSE140 cells. (D) RT‐qPCR was utilised to quantify ZNF468 mRNA expression in KYSE510 cells subjected to AKT activation by SC79, AKT overexpression or inhibition by MK2206 (*n* = 3 biological replicates). Statistical significance was determined using Student's *t* test; ns indicates not significant. (E, F) KYSE510 cells with HA‐ZNF468 expression were exposed to SC79 or MK2206 for 3 h before cycloheximide (20 μg/mL) administration at specified time points. HA‐ZNF468 band intensity was normalised against GAPDH levels and then to the baseline expression under each treatment (Time = 0 min). (G–I) AURKA promoter‐driven dual‐luciferase assays in KYSE510 cells were conducted (*n* = 3 biological replicates). HA‐ZNF468‐expressing cells were treated with SC79 alone or in combination with MK2206 (G) and AKT‐overexpressing KYSE510 cells were transfected with control or shZNF468 constructs (H). HA‐ZNF468‐expressing cells were also treated with or without MK2206 (I). Results are displayed as mean ± SD, with *p* values calculated using One‐way ANOVA; ns: not significant, *p < 0.05, **p < 0.01, ***p < 0.001, ****p < 0.0001.

Previous experimental data have confirmed that ZNF468 promotes the phosphorylation of PI3K/AKT through AURKA, and numerous studies have reported that AKT, once activated, can phosphorylate downstream targets, including transcription factors [13, 14, 15, 16]. Therefore, we considered whether ZNF468 protein expression is regulated by the phosphorylation of AKT. SC79, a unique AKT activator, induces AKT activation within the cytosol and prevents AKT from translocating to the cell membrane by selectively binding to the pleckstrin homology domain [31, 32]. Immunoblotting analysis revealed that SC79 (10 μM) stimulated AKT activity, coincident with an elevation in HA‐ZNF468 protein levels, while treatment with the pan‐AKT inhibitor MK2206 (2 μM) markedly reduced HA‐ZNF468 protein levels (Figure 6A,B). Elevated expression of AKT resulted in increased levels of endogenous ZNF468 protein in both KYSE510 and KYSE140 cells (Figure 6C). Notably, changes in AKT expression or activity had no impact on the ZNF468 mRNA levels (Figure 6D), suggesting AKT primarily regulates ZNF468 protein levels through post‐translational modifications. To further investigate, we assessed whether AKT affects the stability of ZNF468 protein. CHX chase assay revealed that treatment with the AKT activator SC79 extended the half‐life of ZNF468 protein. In contrast, inhibition of AKT activity using MK2206 led to a reduction in ZNF468 protein levels (Figure 6E,F). These findings indicate that AKT enhances ZNF468 protein expression by promoting its stability. Additionally, we explored whether AKT affects the transcriptional activity of ZNF468. Our previous study demonstrated ZNF468 binds to the promoter of AURKA. In line with this, our luciferase assay demonstrated the activation of AKT with SC79 increased AURKA promoter activity, while inhibition of AKT with MK2206 reduced this activity (Figure 6G). Moreover, overexpression of AKT augmented AURKA promoter activity, an effect that was abolished upon knockdown of ZNF468 (Figure 6H). On the other hand, overexpressing ZNF468 significantly boosted AURKA promoter activity, while AKT inhibition with MK2206 reduced the transcriptional activity of ZNF468 (Figure 6I). The findings indicate that AKT not only increases the protein stability of ZNF468 but also amplifies its transcriptional activity on the AURKA promoter.

### 3.8 ZNF468 Is a Phosphorylation Substrate of AKT Kinase and Facilitates AKT Nuclear Translocation via AURKA

**FIGURE 7:**
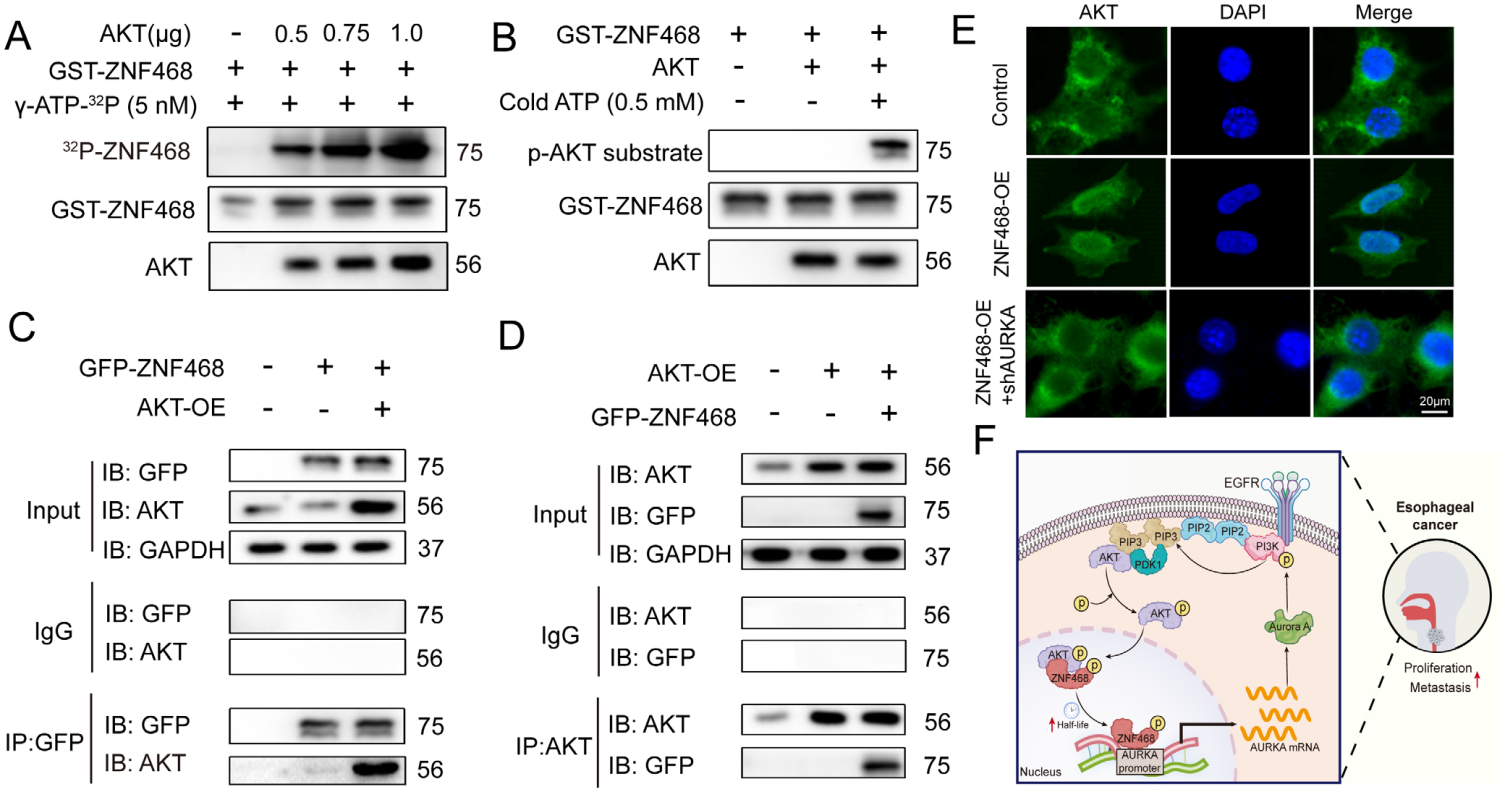
ZNF468 is a phosphorylation substrate of AKT kinase and facilitates AKT nuclear translocation via AURKA. (A) The kinase activity of AKT protein at various concentrations (0.5 μg, 0.75 μg and 1.0 μg) towards GST‐tagged ZNF468 was assessed using a ^32^P isotope radiolabelling method in vitro, co‐incubated with γ‐ATP‐32P (5 nM). The expression levels of ^32^P‐ZNF468 were measured by autoradiography, whereas the expression of GST‐ZNF468 and AKT was confirmed by Western blot. (B) A non‐radioactive in vitro kinase assay was employed to assess the phosphorylation of GST‐ZNF468 by AKT (0.5 μg), co‐incubated with cold ATP (0.5 mM). The protein expression levels of pan‐phospho‐AKT substrate, GST‐ZNF468 and AKT were determined by Western blot. (C, D) The interaction between ZNF468 and AKT was investigated using immunoprecipitation (IP) and reverse IP analyses. ZNF468 and AKT were immunoprecipitated from the total cell lysate of KYSE510 cells overexpressing GFP‐ZNF468 and/or AKT. (E) The expression of AKT protein was assessed by immunofluorescence under conditions of ZNF468 overexpression and combined AURKA knockdown. AKT was visualised with green fluorescence, and the cell nucleus was stained with DAPI (*n* = 3 biological replicates). Scale bar: 20 μm. (F) Schematic of the mechanism pattern.

To determine whether AKT phosphorylates ZNF468, we conducted a radioactive in vitro kinase assay using recombinant AKT and GST‐tagged ZNF468 purified from *E. coli* . Autoradiograph results showed ^32^P‐ZNF468 expression increased with AKT concentration, indicating dose‐dependent phosphorylation by AKT (Figure 7A). Furthermore, using a pan‐phospho‐AKT substrate antibody (Ser/Thr) in a non‐radioactive in vitro kinase assay, we detected phosphorylated GST‐ZNF468 (Figure 7B). We then transfected cells with GFP‐tagged ZNF468 or AKT and conducted immunoprecipitation (IP) and reverse IP experiments, demonstrating their interaction in KYSE510 cells (Figure 7C,D). Using immunofluorescence, we found that overexpression of ZNF468 did not significantly alter AKT expression but increased its nuclear translocation compared to controls. However, this effect was significantly reversed upon AURKA knockdown, suggesting that ZNF468 primarily promotes AKT nuclear translocation via AURKA (Figure 7E). These findings show that ZNF468 is a phosphorylation substrate of AKT and promotes AKT nuclear translocation via AURKA, further elucidating the activation mechanism of the ZNF468–AURKA axis on PI3K/AKT (Figure 7F).

## 4 Discussion

ESCC is one of the main histological subtypes of oesophageal cancer, typically occurring in the middle to upper sections of the oesophagus and originating from the squamous epithelial cells lining the oesophagus [33, 34]. The unfavourable prognosis of ESCC is partly attributed to factors such as tumour cell proliferation, anti‐apoptotic capacity, invasive and metastatic potential and EMT, which are key contributors to the poor outcomes in ESCC.

Our previous study identified the transcription factor ZNF468 as upregulated in ESCC, facilitating radiotherapy resistance by promoting AURKA transcription [5]. However, the role and mechanisms of the ZNF468–AURKA axis in ESCC development and progression remain to be elucidated. Aurora A, encoded by the AURKA gene, is a well‐established oncogenic protein implicated in various cancers, including ESCC [35, 36, 37]. AURKA has been implicated in a range of biological processes that contribute to tumorigenesis, with a particular role in cell cycle regulation, promotion of genomic instability and participation in EMT [38]. The function of AURKA in ESCC has garnered significant attention. Multiple bioinformatics and clinical correlation research has revealed that Aurora A is markedly upregulated in ESCC compared to adjacent non‐cancerous tissues and plays a role in the oncogenic progression of ESCC, making it a potential diagnostic and prognostic marker [37, 39, 40]. On a molecular level, Aurora A directly binds to β‐catenin and promotes its phosphorylation at Ser552 and Ser675, thereby facilitating the malignant progression of ESCC [41]. Additionally, Aurora A enhances the expression of MMP2, which in turn activates the AKT–NF‐kappaB pathway, promoting the invasion and metastasis of ESCC cells [42, 43]. Recent studies have also revealed that Aurora A confers resistance to ferroptosis inducers in ESCC cells [44]. Our research indicates that Aurora A serves as a substantial downstream target of ZNF468, and elevated ZNF468 promotes ESCC cell proliferation, anti‐apoptosis, invasion and EMT partly by upregulating AURKA.

The interaction between AURKA and the PI3K/AKT pathway is also a research hotspot [18, 28]. In ESCC cells, AURKA phosphorylates SDCBP, inhibiting its ubiquitination and degradation, thereby maintaining SDCBP protein stability. SDCBP, in turn, engages directly with EGFR, preserving its localisation to the membrane and triggering the EGFR‐PI3K‐AKT signalling cascade [18]. In a breast cancer study, researchers using kinome‐rewiring techniques discovered that AURKA inhibition reduced the efficacy of PI3K inhibitors [28]. Additionally, research also indicates that AURKA stimulates the EMT and the spread of tumours, specifically in cancers of the stomach and larynx, through the activation of the PI3K/AKT signalling cascade [45, 46]. These studies provide a solid foundation for understanding how AURKA activation of the PI3K/AKT pathway mediates pro‐cancer phenotypes. Our study shows that ZNF468 mediates the oncogenic phenotypes of ESCC via the PI3K/AKT pathway. Treatment with the PI3K inhibitor LY294002 markedly reduced the oncogenic effects of ZNF468 overexpression, indicating that the ZNF468‐AURKA axis promotes tumorigenesis and EMT in ESCC via PI3K/AKT pathway activation.

Overall, this investigation explored the interplay between the ZNF468‐AURKA axis and the PI3K/AKT signalling cascade in ESCC. We proved that ZNF468, through facilitating AURKA transcription, activates the PI3K/AKT pathway, promoting cell proliferation, migration, invasion and EMT. Furthermore, AKT augments the protein stability and transcriptional activity of ZNF468 on AURKA, thereby establishing a positive feedback loop among AKT, ZNF468 and AURKA. Our research findings may offer novel perspectives on the function of the ZNF468‐AURKA axis in ESCC and identify potential therapeutic targets within the PI3K/AKT pathway, offering a promising avenue for developing targeted therapies to combat this aggressive cancer.

While our study provides novel insights into the ZNF468‐AURKA axis in ESCC, limitations warrant further investigation. Our findings are based on a limited number of cell lines, and the generalisability across other ESCC cell lines requires further exploration. Additionally, this study focused on tumour cells and did not explore the role of ZNF468 in the tumour immune and stromal microenvironment, which is an important direction for future research. Furthermore, this study concentrates on molecular regulatory mechanisms at the genetic level within tumour cells themselves. However, it is crucial to recognise that the initiation, progression, metastasis and therapeutic resistance of tumours are also profoundly influenced by complex factors within the tumour microenvironment (TME) [47]. This TME consists of dysfunctional cell populations and is characterised by extensive heterogeneity, including highly variable immune cell infiltration [48]. Therefore, a focus limited to tumour‐intrinsic changes is insufficient to elucidate the underlying mechanisms that support malignant dissemination and metastasis.

## Author Contributions

**Ge Bai:** conceptualization (equal), data curation (equal), formal analysis (equal), investigation (equal), software (equal), visualization (equal). **Lei Wang:** data curation (equal), investigation (equal), resources (equal), software (equal). **Li Zhang:** data curation (equal), formal analysis (equal), investigation (equal), supervision (equal), validation (equal), visualization (equal). **Mayinur Eli:** conceptualization (equal), data curation (equal), supervision (equal), validation (equal), writing – original draft (lead), writing – review and editing (lead).

## Ethics Statement

This study was approved by the ethics committee of the First Affiliated Hospital of Xinjiang Medical University (approval no. 20220308‐091 and 20220309‐106). Written informed consent was obtained from all patients participating in the present study. All the animal experiments were conducted in strict accordance with the guidelines of the Committee for the Care and Use of Laboratory Animals.

## Consent

The authors have nothing to report.

## Conflicts of Interest

The authors declare no conflicts of interest.

## Supporting information


**Figure S1** ZNF468 is upregulated in oesophageal cancer tissues compared to adjacent non‐cancerous tissues, correlates with poor clinical features and is significantly positively associated with Aurora A expression. (A) ZNF468 mRNA levels are elevated in cancer tissues compared to adjacent non‐cancerous tissues across three independent cohorts (GSE53622, GSE53624, TCGA‐ESCA). *T* test or Wilcoxon test. (B) Elevated ZNF468 mRNA expression is associated with higher pathological grades and lymph node infiltration in the TCGA‐ESCA cohort. Kruskal test. (C) AURKA is significantly upregulated in tumour tissues across multiple datasets (GSE104958, GSE130078, GSE52622, GSE53624, TCGA‐ESCA). *T* test or Wilcoxon test. (D) Representative immunohistochemical staining images of ZNF468 and Aurora A in tissue samples from patients with ESCC. Scale bar: 50 μm and 25 μm. (E) Analysis of the differences in mean optical density values of ZNF468 and Aurora A between cancerous and adjacent non‐cancerous samples. Mann–Whitney test. **p < 0.01, ***p < 0.001. Statistical data were presented as mean ± SEM. (F) Pearson correlation analysis indicated a strong positive correlation (*R* = 0.53) between ZNF468 and Aurora A protein expression.

## Data Availability

The data generated or analysed during this study are available from the corresponding author on reasonable request.
