# Supplementary figures and images for: ZNF468/AURKA/PI3K/AKT Positive Feedback Loop Promotes Proliferation and Metastasis of Oesophageal Squamous Cell Carcinoma
Source: J Cell Mol Med. 2025 Jul 24;29(14):e70724. doi: 10.1111/jcmm.70724 (PMC12287617; doi:10.1111/jcmm.70724)

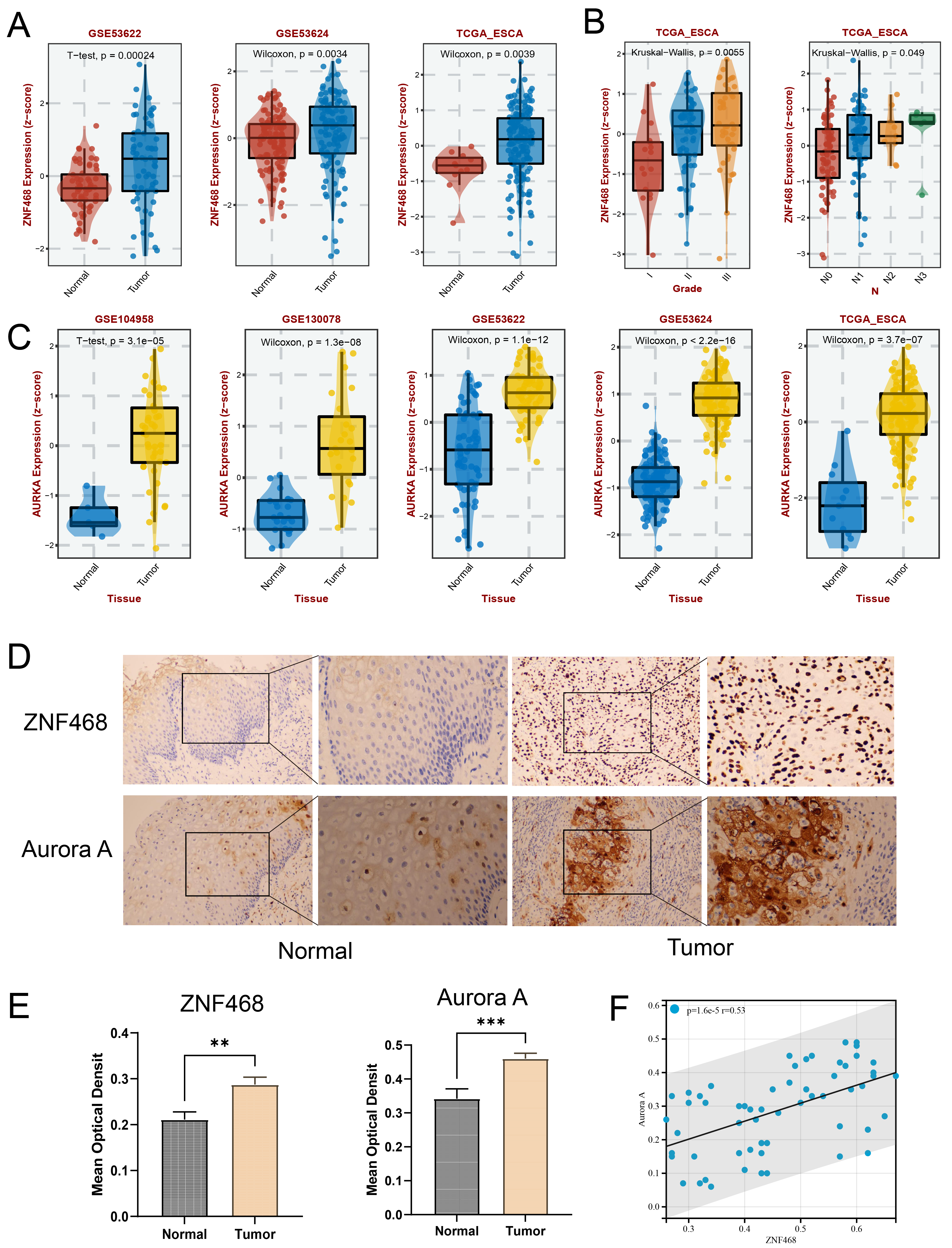

Supplement: Supplementary file 1 — Figure S1 ZNF468 is upregulated in oesophageal cancer tissues compared to adjacent non‐cancerous tissues, correlates with poor clinical features and is significantly positively associated with Aurora A expression. (A) ZNF468 mRNA levels are elevated in cancer tissues compared to adjacent non‐cancerous tissues across three independent cohorts (GSE53622, GSE53624, TCGA‐ESCA). T test or Wilcoxon test. (B) Elevated ZNF468 mRNA expression is associated with higher pathological grades and lymph node infiltration in the TCGA‐ESCA cohort. Kruskal test. (C) AURKA is significantly upregulated in tumour tissues across multiple datasets (GSE104958, GSE130078, GSE52622, GSE53624, TCGA‐ESCA). T test or Wilcoxon test. (D) Representative immunohistochemical staining images of ZNF468 and Aurora A in tissue samples from patients with ESCC. Scale bar: 50 μm and 25 μm. (E) Analysis of the differences in mean optical density values of ZNF468 and Aurora A between cancerous and adjacent non‐cancerous samples. Mann–Whitney test. **p < 0.01, ***p < 0.001. Statistical data were presented as mean ± SEM. (F) Pearson correlation analysis indicated a strong positive correlation (R = 0.53) between ZNF468 and Aurora A protein expression. [file JCMM-29-e70724-s001.tif]
